# Time management practice and associated factors among employees working in public health centers, Northwest Ethiopia: a mixed method study

**DOI:** 10.1186/s12913-023-10004-w

**Published:** 2023-10-24

**Authors:** Sisay Terefe, Lake Yazachew, Desale Bihonegn Asmamaw, Tadele Biresaw Belachew, Amsalu Feleke, Tesfahun Zemene Tafere, Ali Yimer, Wubshet Debebe Negash

**Affiliations:** 1 Department of Health Systems and Policy, Institute of Public health, University of Gondar, Gondar, Ethiopia; 2 Department of Reproductive Health, Institute of Public Health, College of Medicine and Health Sciences, University of Gondar, Gondar, Ethiopia; 3 Department of Public Health, College of Medicine and Health Sciences, Woldia University, Woldia, Ethiopia

**Keywords:** Time management practice, Health care workers, Dabat District, Health Center

## Abstract

**Background:**

While progressing towards universal health coverage, poor time management in the healthcare system had significant effect on an individual such as imbalance, job dissatisfaction, and work ineffectiveness and finally poor productivity of the organization will be resulted. Information about time management practice in the healthcare system is limited. Therefore, the objective of this study was to assess time management practice and associated factors among employees working in public health centers, Dabat District, Northwest Ethiopia.

**Methods:**

A facility-based cross-sectional mixed methods (quantitative and qualitative) study was conducted in Dabat District from May 27 to June 22, 2022. A simple random sampling technique was used to select 413 study subjects and for the qualitative data, six key informants were selected. Self-administered questionnaire was used for the quantitative study, and an interview guide was employed for the qualitative study. Epi-data version 4.6 and SPSS 26 software were used for data entry and analysis, respectively. Open Code 4.6 software was used for qualitative data analysis. Variables with p-value of < 0.05 in multivariable analysis were considered as significant associated factors.

**Results:**

A total of 396 employees participated in the study with a response rate of 95.8%. The result showed that overall, 54.8% (95% CI: 49.5–59.6) of health employees had practiced good time management. The likelihood of good time management was higher among those health workers who had planning experience (AOR = 2.04, 95% CI: 1.22–3.4), low procrastination habit (AOR = 1.65 95% CI: 1.04–2.65), satisfied with performance appraisal (AOR: 1.7, 95% CI: 1.05–2.81), and satisfied with organizational policy and strategy (AOR: 2.6, 95% CI: 1.6–4.3). The qualitative result also showed that the existing performance appraisal practices were not linked to rewards or sanction planning.

**Conclusion:**

The overall time management practice of public health center employees was low compared with prior studies. Organizational policies, prior planning experience, procrastination, and performance appraisal were all significantly associated factors with time management practice. Therefore, health center managers need to set an intervention to address all of the following factors to enhance employees’ time management skills at public health centers like evidence-based performance appraisals, sharing organizational policies, and engaging in capacity building activities such as training in time management and planning.

## Background

Regardless of geographic location, time is a nonrenewable resource [1, 2]. The modern concept of time management entails doing things effectively and efficiently [3]. In the current health care system, time management is a key issue [4]. Planning, assigning, setting goals, delegation, time analysis, monitoring, organizing, scheduling, and prioritizing are all tasks that fall under the umbrella of time management [1].

All health professionals such as midwives, nurses, pharmacists, laboratory technicians, health officers, and health extension workers must be able to manage their time effectively. Inefficient time management can lead to a decrease in the quality of treatment [5]. It is obvious that the goal of appropriate time management at work entails producing high-quality work rather than a large amount of it [6]. Setting goals and priorities as well as scheduling and delegating activities, can help more productive and successful at work, results in an increased job effectiveness, balance, and satisfaction [7].

Currently, many institutions are operating without a time management program, and they misinterpreted it as working for a long time [8]. There is a lack of time management culture in Africa, which can be harmful to both personnel and organizations [9]. Most people believe they have too much to do, not enough time, and they attribute their unmet goals, poor performance, and low productivity to a lack of time [10]. In a healthcare facility, time management is critical since the quality of healthcare service provided to patients can make or break their lives [11].

Quality health care service increases the chance of desired health outcome and is characterized by seven quantitative characteristics, one of which is timeliness. Health centers are organizations tasked with providing routine healthcare services in specified locations. Employees at the health center would be unable to meet all of the expectations that would affect patient care. It can also have negative consequences such as lengthening deadlines missed a significant level of tension, increasing treatment expenses, low work quality, inefficient systems, violating patient rights, and disturbing the work of other teams as well as a negative impact on the careers path [12].

There is a difference in the degree of time management from country to country; it is 69.5% in Palestine, 49.5% in Iran, 53.7% in Ghana, and 56.4% in Ethiopia [13–16]. A study conducted in South Africa revealed that nurses in the field have inadequate time management skills. In an Egyptian investigation on the association between time management and physical stress symptoms, over two-thirds of the study sample spent more time on useless nursing duties than on productive nursing duties [17]. As a result, both patients and healthcare providers’ ideal time must be factored into healthcare provision and usage [18]. Improvement in healthcare delivery necessitates a purposeful focus on service quality, which entails providing timely, equitable, integrated, efficient, effective, safe, and health services [19]. The devastating consequences of low time management practice are poor professional reputation, inefficient workflow, uncontrollable stress, poor work quality and professional decay [16].

**Fig. 1:**
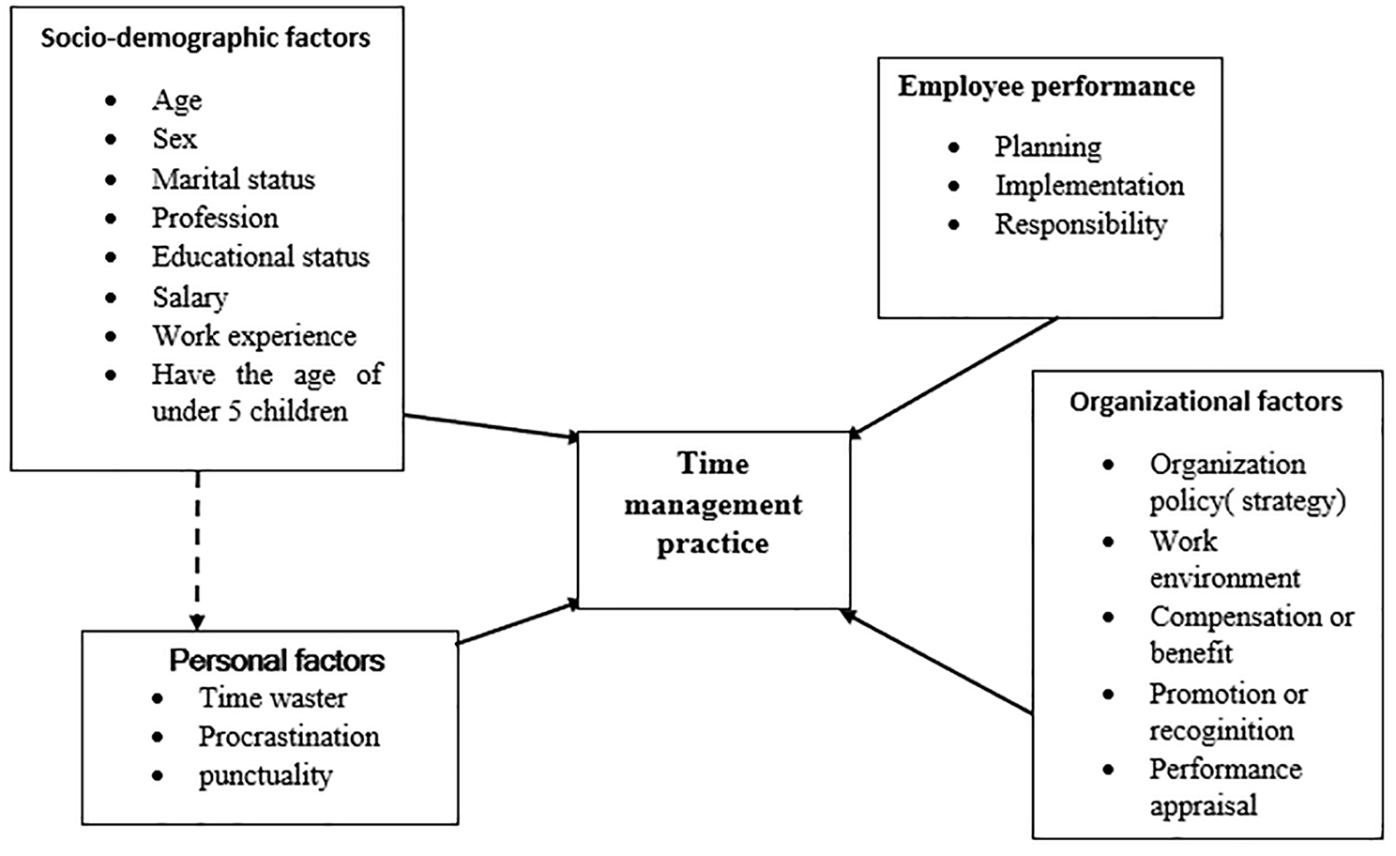
Conceptual framework of factors affecting employees’ time management practices at Dabat District northwest Ethiopia, 2022. Adapted from different of literature [7, 14, 20]

There are different factors which could affect time management practice. These factors are personal factors like procrastination habit, punctuality and time waster [14], organizational factors like organizational policy, performance appraisal, work environment, compensation to benefit promotion or recognition and employee performance related factors like planning experience, responsibility and implementation [7, 14, 20]. Other socio-demographic factors such as age, profession, marital status, educational status, salary, experience and sex have also been considered as associated factors [15, 21, 22]. In addition, administrative and managerial barriers are common issues that affect time management up 69.7% of the total [23]. The factors associated with time management practice are summarized in the conceptual framework (Fig. 1).

Health center employees are among the most vital resources of the health care delivery system; a committed health center employee has great importance for an organization and population. The better quality of health care resulting from a punctual and committed health workforce could benefit the individual patient and the community at large. TMP (time management practice) in every organization particularly health system organization is still the neglected problem which is the bottleneck for the success of employees.

Even though there are limited studies conducted to assess time management practice, almost all of them were concentrating on the educational sector [24–26] and in primary hospital [27]. Moreover, most of the previous studies used only quantitative method which makes the result weak and less informative. Health centers are always existed as the description of the point of first contact between patients and the health care system [28]. In these key facilities, time management is a multi-dimensional problem which is difficult to address only by a quantitative approach but requires both a quantitative and qualitative approach. The findings might be used to improve TMP among health center workforce by developing an approach to solve obstacles through effective time management practice. It also enables health facility manager to prevent poor health service quality.

As a result, this research is expected to fill the aforementioned research gaps by a cross sectional mixed study design. Adopting a mixed-methods approach adds value to the study by presenting comprehensive quantitative and qualitative findings and factors influencing time management skills among employees. Therefore, this study aims to assess TMP and associated factors among employees working at Public Health Centers of Dabat District, North West, Ethiopia.

## Methods

### Study settings, design and period

Institutional based cross-sectional mixed study design was conducted from May 27 to June 22, 2022. The specific type of mixed study design used in this study was sequential explanatory design. Hence, the quantitative data was collected first, then to elaborate the quantitative findings qualitative data was collected.

Ethiopia’s healthcare service is structured into a three-tier system: primary, secondary and tertiary levels of care. A primary health care unit (PHCU) comprises four health centers (HCs), five health posts within each health center, and a primary hospital. Each health post is responsible for a population of 3,000–5,000 people. A health center provides both preventive and curative services. In addition to what an HC can provide, a primary hospital provides emergency surgical services, including caesarean section and gives access to blood transfusion services. Secondary level of care consists of general hospitals. In addition, it serves as a referral center for primary hospitals. Tertiary level of care comprises federally-run, specialized hospitals and university hospitals. The tertiary level of health care used as a referral center for general hospital and serves 3.5 to 5 million population [29, 30].

This study was conducted at public health centers, in Dabat district, north-west Ethiopia, one of the eight districts that make up the north Gondar Zone. Dabat District is divided into 35 kebeles (the smallest administrative units) and is located in northwest Ethiopia. There is only one primary hospital in the district. Dabat district has six public health centers and 35 health posts. There are 525 employees in the six health centers, of which 330 are health professionals, and 195 are administrative workers. According to data from the Dabat district health department’s plan office, the district’s health facilities serve around 191,977 people.

### Population

The source population of the study were all employees working in Dabat District public health centers. All selected employees who are working in Dabat District public health centers were the study population. All full-time and more than six months served employees in public health centers who were available during the study period were included. On the other hand, those employees who had worked for less than six months in the public health center were excluded from this study. This is because those health workers who had worked for less than six months may not have enough information about the health facility related questionnaire.

### Sample size estimation

The sample size for time management practice was determined by using a single population proportion formula by considering an assumption of the proportion of TMP 57.1% [31] a 5% margin of error, a 95% confidence level, and a 10% non-response rate. The sample size for the first objective was calculated using a single population proportion formula as follows.

n =(z_α/2_)^2^ p (1-p) /d^2^ n = (1.96)^2(^0.571)(0.429)/0.05^2^.

z_α/2_=confidence interval at 95% n = 376.

d = level of significance at 5%.

p = population proportion 57.1%.

When added 10% non-response rate = 37.

The total sample size was 376 + 37 = 413 whereas, sample size for the independent variable using double population proportion formula described in Table 1 below.

**Table 1:** Sample size calculation for associated factors by using two population proportions among employees working in public health centers of Dabat District North West Ethiopia, 2022

Factors	Confidence Interval at	Power(1-β)	Ratio	The proportion of TMP among the Exposed group	The proportion of TMP among the non-exposed group	Sample size	
						n	Adding 10% non-response rate
Performance appraisal	95%	80%	1	39.8	72,9	162	178
Organizational policy and strategy	95%	80%	1	40.7	74.9	76	83
Procrastination	95%	80%	1	38.3	69.06	94	103

Finally, to conduct this study, the maximum sample size of 413 was revealed from the single population proportion result and used for this study.

### Sampling technique and procedure

#### Quantitative part

**Fig. 2:**
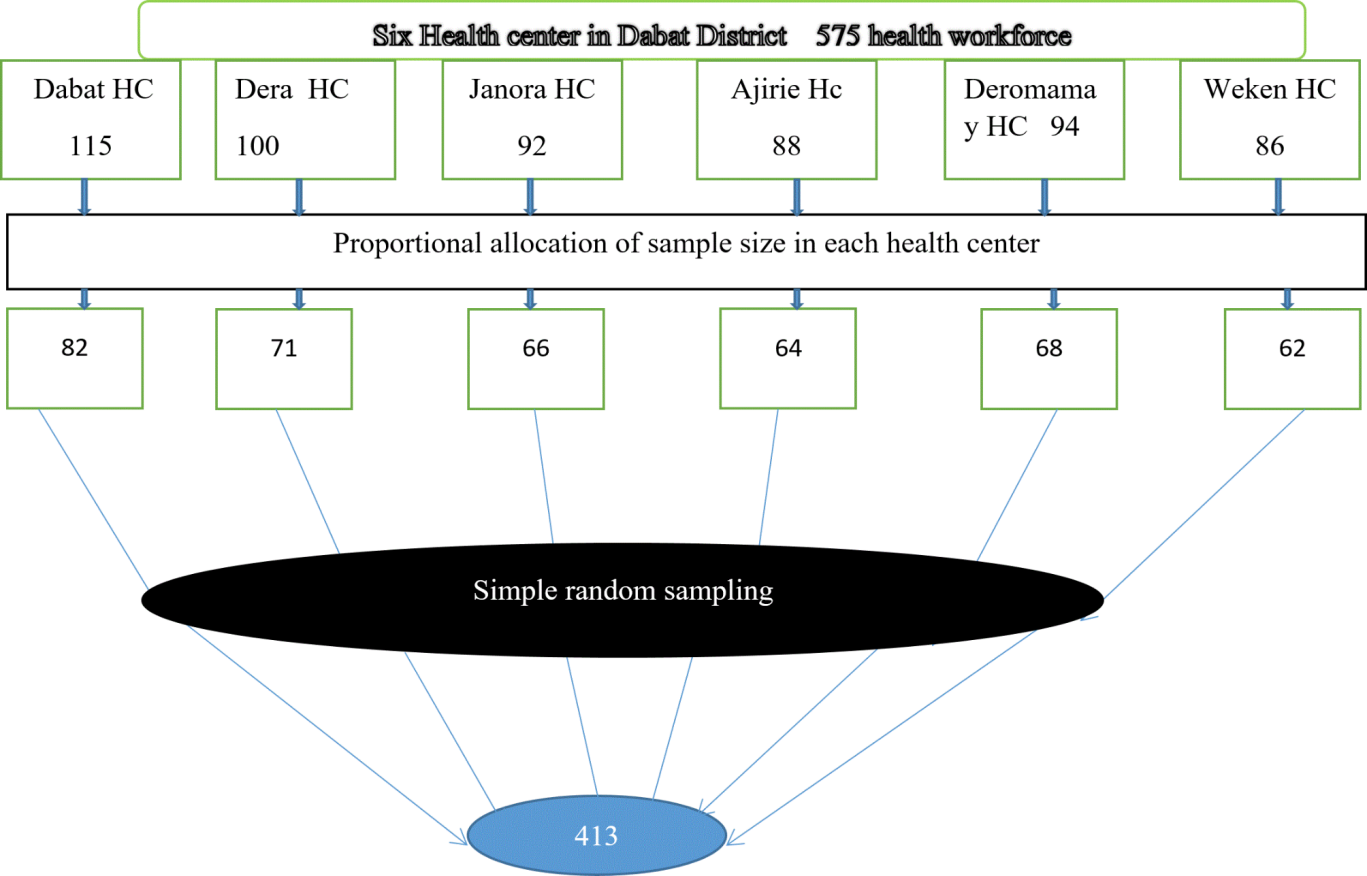
Schematic presentation of the Sampling procedure for TMP and associated factors in the public health center of Dabat district Northwest Ethiopia

A simple random sampling technique was used to choose the sample from six health center employees’ payrolls. Human resource management was utilized as a frame for each employee, and then the number of health center employees was proportionally assigned to the sample size to choose 330 health professionals and 195 administrative workers from Dabat District. Finally, a simple random sampling procedure was used to choose each participant from a pool of 575 public health center employees based on the proportional allocation formula for the quantitative data (Fig. 2).

For the qualitative study, purposive sampling was used to select five participants. Health center managers were selected for key informant interviews since they have direct work relations with the study objectives.

### Variables and measurement

#### Dependent variable

For this study, time management practice is scheduled use of time by employees. It was assessed by eight items measured by five point Likert scale, with one denoting strongly disagree and five denoting strongly agree. After dichotomous category responses, above a score of 65% had categorized as a good time management practice [14, 32]. Independent variables operational definition is summurized under the following table (Table 2).

**Table 2:** Independent variables and measurement for time management practice

Variables	Descriptions
**Compensation**	Compensation was assessed using three items, about employees’ feelings of fairness and proper compensation for their labor, as well as financial incentives for higher performance. Each of which was graded by a 5-point Likert scale. finally, it was grouped as satisfied if their responses were greater than the mean score value and unsatisfied if their answers were less than the mean score value [27].
**Organizational policy**:	Organizational policies and strategies were used to express the respondent’s feelings about the implementation of organizational policies and strategies. It was evaluated using three items, each of which was graded by a 5-point Likert scale. finally it was categorized as satisfied and unsatisfied based on the mean value [31]
**Time waster**	Workers who engage in different activities or things that consume lots of their time without offering any productive outcome. The variable was measured by five items having five point Lickert scale for each item. The responses were classified as “low” if they were greater than the mean score value, and as “high” if they were less than the mean score value [31]
**Performance appraisal**	Performance appraisal was evaluated using three items about the participants’ feelings of how their real performance with a 5-point Likert scale. This was categorized as satisfied if their responses were greater than the mean score value; they were labeled as unsatisfied if their responses were less than the mean score value [14]
**Procrastination**	Workers considered the tendency to delay intended actions and delay scheduled work are referred to as procrastinators. It was assessed using four items, each of which was graded on a five-point Likert scale. If the responses are larger than the mean score value, the variable was classified as “low,“ and less than the mean score value, they were classified as “high.“ [33]
**Technical staff**	For this study technical staff were considered the midwives, nurses, pharmacists, laboratory technicians, health officer, health extension workers, and health information workers
**Non-technical staff**	Those employees who work as health center administrative workers and are not part of the health professional staff were considers as non-technical staff.

### Data collection tool and procedures

#### Quantitative data collection

The data collection tool for the quantitative study was a self -administered questionnaire. The instrument was adapted from previously published literature [14, 31]. The questionnaire was designed in English translated to the local Amharic language, and then returned to English by the third person to check for consistency. The questionnaire has a total of 47 questions divided into three parts; the first was questions assessing personal factors related, the second part organizational factor-related questions and the third part included questions on employee performance factors related questions. Five licensed diploma nurses and three B.Sc. nurse supervisors participated in the data collection. To ensure that each employee of the health center employee received the same instructions and information, a written guideline was provided to the supervisor of administration of the questionnaire. After ensuring the study population’s willingness to participate, the instruments were distributed among individuals.

### Qualitative study

The qualitative data were collected using a semi-structured interview guide containing questions, and an interview guide containing three questions written in English and translated into Amharic. A semi-structured interview guide questionnaire created using key informant interviews performed in English and translated to Amharic was used to collect the qualitative data from five health center managers. One key informant was excluded from the study for security reasons. The interview was recorded, and the principal investigator made a note of the key points. An average of 30 min was allocated for each interview.

### Data quality control

The questionnaire was prepared in English first, then translated into Amharic and back to English to ensure consistency. The self-administered survey was pre-tested using 5% of the sample size before the data collection period at Debark City health center. Next to the pretest, the questionnaire was revised, checked for problems like unclear language and unclear questions. While selecting data collectors and supervisors based on their research skills, the principal investigator offered a one-day training session on the study’s objectives, instruments, and data gathering techniques. The instruments were also evaluated by experts in public health research. Each data collector checked every questionnaire for completeness. Every day, the supervisors and principal investigator had to examine and check each questionnaire for accuracy and completeness. Cronbatch alpha was done to check the internal consistency of the tools, and each independent variable item scored from 0.7 to 0.9. Whereas for the dependent variable it was found 0.772.

### Data processing and analysis

#### Quantitative analysis

After a consistency and completeness check, the data were combined and coded. It was finally entered into EPI-data version 4.6. After reviewing the original data, errors were corrected using the code numbers assigned to the surveys. The entered data was exported to the statistical software SPSS version 26 for analysis. To obtain consistent effects of measurement items, reverse coding was performed for negative questions. Frequencies, percentages, and cross-tabulations were used to summarize the descriptive statistics of the data. A reliability test (Cronbach’s alpha) was conducted to assess the questioner item’s dependability. Bivariable and multivariable logistic regression models were used to identify characteristics that influence time management practice. Variables with a p-value < 0.2 in the bivariable analysis were included in the multivariable analysis, and variables with a p-value < 0.05 in the multivariable analysis were regarded as significantly associated factors. Finally, the variable with the highest odds ratio (AOR), a 95% confidence interval and a significance level of 0.05 was used to find the strongest link to time management practice. The overall TMP’s Hosmer and Lemeshow goodness of fit had a p-value of 0.766 as well. In addition to checking for collinearity between independent variables, multi-colinearity diagnostic test was performed (mean VIF result = 1.4).

#### Qualitative study

Amharic language was used to record the interview information. After the audio was transcribed, it was translated into English to make sure the information acquired by Amharic language experts was precise and consistent. To ensure trustworthiness, the data was collected from each health facilities, developing a process for analyzing the data, recruiting and retaining qualified individuals to complete coding and initial analysis, and completing in-depth analysis of the data. A transcription of the data was made the day after it was collected, and then an analysis was performed. Secondly, there was a thorough and exact examination of each transcribed verbatim line by line to establish initial codes, which were then categorized based on the similarity in meaning. Prior to interviewing the next participant, we cleaned the data by eliminating all distinguishable information. On the basis of the categorized data, the researchers developed sub-themes and themes. They grouped the codes with associated meanings into one group called sub-themes and considered their significance. Afterwards, all codes and categories were categorized into themes (Table 3).

**Table 3:** thematic analysis for time management practice among public health center employees in Dabat district Ethiopia

	Category	Subcategory		
1	Organizational factors	1.1Working environment	1.2 Compensation	1.3 Recognition, promotion
2	Personal factors	2.1 Attitude	2.2 Motivation	2.3 Socialization
3	Employees performance factors	3.1 Planning	3.2 BSC result	3.3 Feedback

## Results

### Socio-demographic characteristics of the respondents

A total of 396 (95.8%) of the health workers participated in the study. The average mean age of participants was 29.59 (SD ± 5) years. More than half (52%) of the participants were married, and 59.8% were female. Regarding the educational status of the study participants, 51% had diploma. Additionally, 71% of the participants who were working in the study area were technical staff (Table 4).

**Table 4:** Socio demographic characteristics of employees working in public health centers of Dabat District Northwest Ethiopia, 2022 (n = 396)

Variable	Category	Frequency (n = 396)	Percentage (%)
Age (years)	20–25	90	22.8
	26–30	178	44.9
	Above 30	128	32.3
Sex	Female	206	52.
	Male	190	48.0
Marital status	Single	146	37
	Married	237	59.8
	Divorce	13	3.3
Educational status	Diploma and below	201	50.8
	Degree	180	45.5
	masters and above	15	3.8
Work experience in year	1–5	200	50.5
	6–10	103	26.0
	More than 10	93	23.5
Salary (ETB)	Below 6193	259	65.4
	6193–8017	107	27
	Above 8017	30	7.6
Profession	Technical	281	71
	Non-Technical	115	29
Had under five aged children	No	245	61.9
	Yes	151	38.1

### Personal-related characteristics of the respondents

As shown in Table 5, two hundred seventeen (54.8%) of workers had a high procrastination habit, and 223 (56.3%) had a high time-wasting work habit. This result is similar to the qualitative findings that showed time management practice depends on the habit and attitude of the workers for their value towards time management.

A 34 years old health center manager said that “I want to express my concern regarding employee time management restrictions, which I believe are negative issues, such as failing to finish assignments on time, wasting time on social media, and attending needless meetings. These factors affect employees’ ability to manage their time effectively.”

**Table 5:** Personal related factors of respondents working in public health centers of Dabat District Northwest Ethiopia, 2022 (n = 396)

Variable	Category	Frequency	Percentage (%)
Procrastination	High	217	54.8
	Low	179	45.2
Time waster	High	223	56.3
	Low	173	43.7
Punctuality	No	185	46.
	Yes	211	53.3

#### Organizational-related factors of TMP

The result showed that two hundred sixteen employees (54.5%) were dissatisfied with the public health center’s organizational policies. Similarly, 203 (51.3%) of employees were unsatisfied with the performance appraisal systems in their health centers, and the study found that 238 (60.1%) employees were unsatisfied with the health centers’ compensation and benefits packages (Table 6). Similarly, the qualitative findings also showed that the policies and procedures at the health center did not have access for the staff members, and they have dissatisfied with the health center’s performance appraisal systems. Additionally, Employees were not able to receive benefits due to financial constraints, which led to dissatisfaction in the whole environment of the health center.

Although there are policies and procedures in place at the public health center, staff members do not have access to them, are not trained in them, and do not get awards that the policy has approved. As a result of these challenges, employees’ time management has been reduced, and productivity has declined. Performance assessment systems are not strong, and implementation of the BSC system is weak.“ one key informant from the health center manager Said.

Another 29 year old health center manager explained that “Employees are unable to receive benefits due to financial constraints, particularly administrative staff, who are unable to get government benefits on time as a result, negatively affecting their productivity and time management efforts”.

And another 30-year-old manager confirmed, that “I am not satisfied with my working environment in the health center since there is no staff housing nearby, there is a lack of medical supplies and equipment, there is no internet access, and there is a lack of respect for peace and security. All the lists are barriers to providing a pleasant environment for health workers to manage their time efficiently and effectively. The problems have had an impact on employees’ time management practices.“

**Table 6:** Organizational-related factors of respondents working in public health centers of Dabat District Northwest Ethiopia, 2022 (n = 396)

Variable	Category	Frequency	Percentage (%)
Organizational policy	Unsatisfied	216	54.5
	Satisfied	180	45.5
Performance Appraisal	Unsatisfied	203	51.3
	Satisfied	193	48.7
Working environment	Bad	219	55.3
	Good	177	44.7
Compensation or benefit	Unsatisfied	238	60.1
	Satisfied	158	39.9
Recognition or promotion	Unsatisfied	73	18.4
	Satisfied	323	81.6

#### Employees’ performance-related factors

This study revealed that 206 (52%) of health center employees had little experience with work-related planning. According to the results of this study, 173 (43.7%) employees of public health centers took low responsibility for their work. About 48.5% of respondents had not accomplished their work on time (Table 7).

The qualitative finding also supported these findings, which most respondents explained that the existing performance appraisal practices were not linked to rewards or sanctions planning. A key informant said that *“*Existing performance appraisal practices are not linked to rewards or sanctions planning. This is just another decisive factor in effective time management. Without adequate preparation of what to perform at a certain time.”

**Table 7:** Employees performance related factors of respondents working in public health centers of Dabat District Northwest Ethiopia, 2022 (n = 396)

Variable	Category	Frequency	Percentage (%)
Planning	No	206	52.0
	Yes	190	48.0
Implementation	Low	192	48.5
	High	204	51,5
Responsibility	Low	175	44
	High	221	56.0

### Factors associated with time management practice

A bivariable logistic regression model was done to identify possible factors that affect employees’ time management practices in their current workplace. Age, salary, profession, punctuality, organizational policy, performance appraisal, working environment, planning, implementation, responsibility, recognition and promotion, procrastination, and time wasters were all found to be good candidates for multivariable logistic regression analysis. In the multivariable logistic regression analysis, planning experience, organizational policy and strategy, procrastination, and performance appraisal were variables that significantly associated with time management practice among public health center employees.

Correspondingly, Table 8 shows that those employees who were satisfied with organizational policy and strategy were 2.6 times more likelihood to have a good TMP than their counterparts (AOR: 2.6, 95% CI: 1.6–4.3). Employees who were good at planning their jobs, were two times more likely to have good time management practices than those who did not (AOR = 2.04, 95% CI:1.22–3.4). Likewise, respondents with low procrastination habits were 1.65 times more likely to have a good TMP than those with high procrastination habits (AOR = 1.65: 95% CI: 1.03–2.65). Similarly, those employees who had satisfied with performance appraisal were 1.7 times more likely to have good TMP than those employees who had dissatisfied with the public health centers performance appraisal system (AOR = 1.7, 95% CI: 1.05–2.81).

**Table 8:** Birvariable and multivariable logistic regression analysis of factors associated with TMP among employees, Northwest Ethiopia, 2022 (n = 396)

Variable	Category	Time management practice		OR at 95% CI	
		Good	Poor	COR(CI)	AOR(CI)
Age (years)	20–25	40	50	1	1
	26–30	90	88	1,28(0.77–2.1)	1.084(0.60–1.93)
	Above 30	87	41	2.1(1.5–4.63)	2.049(0.56–2.84)
Salary(in ETB)	Below 6193	133	126	1	1
	6193–8017	67	40	1.6(0.44–6.90)	1.28(0.75–2.2)
	> 8017	17	13	1.23(0.65–9.73)	1.51(0.30–7.48)
Profession	Technical staff	148	133	0.74(0.48–1.14)	1.14(0.67–1.94)
	Non-technical staff	69	46	1	1
Procrastination	High	138	79	1	1
	Low	79	100	0.45(0.40–1.30)	1.65(1.04–2.65)**
Time waster	High	147	76	1	1
	Low	70	103	0.35(0.23-0.53)	0.81(1.05–2.80)
Punctuality	No	119	66	1	1
	Yes	98	113	0.48(0.32-72)	0.80(0.49–1.30)
Organizational policy	Unsatisfied	110	70	1	1
	Satisfied	107	109	0.62(0.42-0.93)	2.6(1.60–4.30)*
Working environment	Bad	138	81	1	1
	Good	79	98	0.47(0.31-0.71)	0.07(0.43–1.12)
Recognition and promotion	Unsatisfied	46	27	1	1
	Satisfied	171	152	0.66(0.55–1.23)	0.078(0.40–1.51)
Planning	No	133	84	1	1
	Yes	57	122	0.29(0.0.20-0.0.45)	2.04 (1.22–3.4)**
Performance appraisal	Unsatisfied	81	122	1	1
	Satisfied	98	95	1.55(1.04–2.31)	1.7(1.05–2.81)*
Responsibility	Low	51	124	1	1
	High	128	93	3.34(2.00-5.10)	0.35(0.57–1.50)
Implementation	Low	130	62	1	1
	High	87	117	0.35(0.23–0.53)	0.74(0.44–1.23)

COR; Crude Odds Ratio, CI: Confidence Interval, AOR: Adjusted Odds Ratio, 1: Reference category

* Significant at p < 0.05

** Significant at p < 0.001

### Result summary

In this study a total of 396 (95.8%) health workers participated. Of the participants, 54.8% had high procrastination habit, and 56.3% had a high time-wasting habit. The result is supported by qualitative study as “The attitude of the employees has affected their time management practice which I believe are negative issues, such as failing to finish assignments on time, wasting time on social media, and attending needless meetings. These factors affect employees’ ability to manage their time effectively.” The result showed that 54.5% and 51.3% were dissatisfied with the public health center’s organizational policies and performance appraisal, respectively. “Although there are policies and procedures in place at the public health center, staff members do not have access to them, are not trained in them. As a result of these challenges, employees’ time management has been reduced, and productivity has declined.“ one key informant from the health center manager Said” And another 30-year-old manager confirmed, that “I am not satisfied with my working environment in the health center since there is no staff housing nearby, there is a lack of medical supplies and equipment, there is no internet access, and there is a lack of respect for peace and security. All the lists are barriers to providing a pleasant environment for health workers to manage their time efficiently and effectively. The problems have had an impact on employees’ time management practices.“ In the multivariable regression analysis, planning experience, organizational policy and strategy, procrastination, and performance appraisal were variables that were significantly associated with time management practice among public health center employees.

## Discussion

This study has tried to determine the magnitude and associated factors of TMP among employees working in the public health center of Dabat District Northwest Ethiopia. According to the findings of this study, the overall magnitude of time management practice was 54.8% (95% CI: 49.5 − 59.6). The result was higher than researches conducted in Egypt (45%) [33], Pakistan (30%) [34], and Dire Dawa University, Ethiopia (34%) [35]. On the other hand; the magnitude of TMP among the public health center employees was in line with studies conducted in India (56.29%) [36], Ghana (53.75%) [37], Ethiopia (56.4%) [14]. This consistency may be resulted from selection of study participants from mixed professionals rather than dealing on a single professional alone. Moreover, the higher time management practice might be followed the design of the policies, and action plans implemented by the health centers for the improvement of the health care system across each country.

However, it is lower than studies conducted in Palestine, and the United Arab Emirates where time management practice was (69.5%) [32], (59.7%) [38], respectively. This may be due to differences in health institutions infrastructure, study area and study participants. It might be also due to variation in sample size. Additionally, it was lower than studies done among senior nurses in Istanbul, Turkey (87.8%), and Iran (96.7%) [39]. The lower prevalence of time management practice in our study may be due to the work settings of the study participants. All the previous studies were conducted at hospital level where as the current study was conducted at health center level where there is low resource availability as compared to hospital settings. Additionally, this disparity may also be due to the homogeneity of the study participants’ senior nurses alone in Iran, nurses and midwives in Istanbul and the study subjects in Palestine were health professionals who worked in both public and private hospitals. There were also differences in study design, the study area, study participants, health institutions’ infrastructure, advancement of technology, health workforce attitudes due to diverse cultures, values, and beliefs, management practice, and organizational policies and guidelines. To achieve effective time management, managers and employees of public health centers must discuss about setting goals, prioritizing actions, and scheduling activities for implementation.

This study pointed out that workforces who were satisfied with the organization’s policies and strategies were efficient time managers as compared to their counterparts. This is supported by research conducted in Palestine [40, 41], Netherlands [42], Egypt [43] and North Gondar (Ethiopia) [14]. If employees feel that the organization’s policies and initiatives do not serve everyone fairly, this may negatively affect how they manage their time. Inversely, satisfied respondents would be motivated and able to manage their time well at work because they think their organizations are fair and that they benefit from them. The positive association between satisfaction and time management may be due to health professionals’ strong attachment to their organization and being satisfied with the policies and strategies of the organization. Moreover, satisfied employees can grow in trust and ownership. All the above-mentioned reasons lead to encourage good time management [44, 45].

Public health center employees who had prior planning experience did well in terms of time management. This result is similar to a study conducted in the United Arab Emirates [38], Winneba, Kumasi city [46], Dire Dawa University, Ethiopia, Nigeria [2, 35]. Similarly, this finding is also supported by the Pareto theory of time management, argues that “there is never enough time to do everything, but there is always time to do the most important things” [47]. The main premise of this principle demonstrates that task prioritizing is the main cornerstone of planning [37, 47]. If personnel were well-organized, they would make an effort to carry out their activities by their plan, directly maximizing their TMP. Strong task preparation experience can potentially lessen an employee’s dependency on task leading and instructions to do. Hence, for effective time management, managers of healthcare facilities should motivate and strengthen health professionals to develop plans and adhere to them.

Additionally, procrastination had a significant association with TMP. Low procrastination habits’ were more likely to have a good TMP than those with high procrastination habits. This result was also supported by a study conducted in Nigeria, which found that most of the health workers spent in hospitals on tedious tasks [44]. This finding is supported by qualitative findings such as failing to finish assignments on time, wasting their time on social media, and attending unnecessary meetings. Most of their time is spent in conversation, attending pointless meetings in their offices and during work hours, and taking unneeded breaks and vacations. If people delay at work, all of their planning, goal-setting, activity prioritization, and other efforts will be nothing [44]. It is implied that health workers are expected to accomplish tasks or to avoid postponing activities as a way of efficiently managing their time.

Moreover, the health center respondents who were satisfied with their performance appraisal results had a greater time management practice than those who were unsatisfied with their performance appraisal results. This finding is supported by qualitative findings; such as there is no connection between current performance review procedures and incentives or penalties. Both the BSC system’s implementation and performance assessment mechanisms are inadequate. A study conducted on the time management skills of academic performers [48] supports this statement. This might be related to how the staff feels about the organization’s operating system. Similar to this study participants who were satisfied with the performance appraisal system in their organization had more likely to have a good TMP than their counterparts [14]. This result was confirmed by Locke’s discrepancy satisfaction theory, which claims that an employee is more likely to be satisfied if there is less of a gap between the job outcomes he receives and what he desires [38]. Additionally, it was consistent with Adam’s equity principle, which argues that employees will be more satisfied if their outcomes to inputs ratio are equivalent to that of other employees [49]. The finding implies that for good time management practice, it is crucial that all performance appraisal indicators must be appropriately designed and agreed upon by all health workers.

The finding of the qualitative result revealed that there is no way for employees to learn about the institution’s policy and not receive benefits in accordance with it, employees are lacking in planning abilities, the BSc evaluation system is inadequate, and failed to finish tasks. All the aforementioned reasons were barriers, which affect time management practice.

### Strength and limitations of the study

As a strength, the study tried to address extensively TMP through incorporating important variables such as procrastination and punctuality which have been missed in previous studies. Additionally, using qualitative and quantitative method makes this study extremely unique. On the other hand, we didn’t separately study TMP across specific health professional’s category which might affect a clearer picture of the relationship between TMP and profession. Use of self-administered questionnaires may have some potential for reporting biases. Assessments of key informants only from the health center manager may not adequate to explore the barriers of poor time management. Cross-sectional study design tells snap-shot information which might not be preferable to TMP like follow up and other study designs.

## Conclusion

When compared to earlier studies, the overall time management practice was low. Organizational policy and strategy, procrastination, performance appraisal, and planning experience were all revealed significantly associated factors with time management practice. Managers at public health centers need to come up with an intervention that takes all of the aforementioned factors into consideration so that employees may better manage their time at work. Health center executives are needed to be skilled in assessing, developing, and implementing organizational rules, policies, and performance appraisal systems that will treat every employee fairly and equitably and improve time management practice. Health center employees are required to discuss with their leader about issues that make them unhappy at work. Health center managers should modify their attitudes about scheduling and plan to the institution’s strategic goal. Finally, future researchers are recommended to fully address the issue that include observational and follow-up study approaches in larger study settings like in health posts and hospitals.

## Data Availability

The datasets used and/or analysed during the current study available from the corresponding author on reasonable request.

## List of abbreviations

CSA: Central Statistical Agency
EPI-INFO: Statistical Packages for Epidemiological Information Analysis
HSTP: Health Sector Transformation Plan
ICU: Intensive Care Unit
SPSS: Statistical Package for Social Sciences
TMP: Time Management Practice
IOM: International Organizational Migration
UoG: University of Gondar
WHO: World Health Organization
